# The strange case of AMPK and cancer: Dr Jekyll or Mr Hyde?^†^

**DOI:** 10.1098/rsob.190099

**Published:** 2019-07-10

**Authors:** Diana Vara-Ciruelos, Fiona M. Russell, D. Grahame Hardie

**Affiliations:** Division of Cell Signalling and Immunology, School of Life Sciences, University of Dundee, Dow Street, Dundee DD1 5EH, UK

**Keywords:** AMPK, cancer, LKB1, metabolism, tumour suppressors, tumour promoters

## Abstract

The AMP-activated protein kinase (AMPK) acts as a cellular energy sensor. Once switched on by increases in cellular AMP : ATP ratios, it acts to restore energy homeostasis by switching on catabolic pathways while switching off cell growth and proliferation. The canonical AMP-dependent mechanism of activation requires the upstream kinase LKB1, which was identified genetically to be a tumour suppressor. AMPK can also be switched on by increases in intracellular Ca^2+^, by glucose starvation and by DNA damage via non-canonical, AMP-independent pathways. Genetic studies of the role of AMPK in mouse cancer suggest that, before disease arises, AMPK acts as a tumour suppressor that protects against cancer, with this protection being further enhanced by AMPK activators such as the biguanide phenformin. However, once cancer has occurred, AMPK switches to being a tumour promoter instead, enhancing cancer cell survival by protecting against metabolic, oxidative and genotoxic stresses. Studies of genetic changes in human cancer also suggest diverging roles for genes encoding subunit isoforms, with some being frequently amplified, while others are mutated.

## 1. Introduction

The AMP-activated protein kinase (AMPK) is best known as a sensor of both cellular [1–3] and whole body [4,5] energy status. AMPK is activated when ATP bound at a key site on its *γ* regulatory subunit is displaced by AMP and/or ADP, causing conformational changes that trigger allosteric activation, as well as promoting net phosphorylation (and consequent activation) of the catalytic subunit by upstream kinases. As ADP rises and ATP falls during situations of cellular energy stress, the reaction catalysed by adenylate kinases (2ADP ↔ ATP + AMP) is displaced rightwards, ensuring that AMP rises to an even larger extent than ADP [6], thus activating AMPK in a very sensitive manner. AMPK is also activated by increases in intracellular Ca^2+^ [7–9], by glucose starvation [10] and by DNA damage [11–13] via non-canonical, AMP/ADP-independent mechanisms. By phosphorylating downstream targets that switch on catabolic pathways, while switching off anabolic pathways and other ATP-consuming processes such as progress through the cell cycle, AMPK not only promotes ATP synthesis but also restricts cell growth and proliferation in an attempt to restore energy homeostasis and maintain cell viability.

Given this propensity to switch off cell growth and proliferation, and the discovery that the principal upstream kinase phosphorylating and activating AMPK was the well-established tumour suppressor LKB1 [14–16], it seemed likely that AMPK would play a beneficial role (Dr Jekyll!) in cancer and act as a tumour suppressor. There is indeed evidence supporting this, at least in some cancer types, as well as for the obvious corollary that AMPK activators should delay tumorigenesis in those cancers. However, there is contrasting evidence that, in other contexts, the presence of AMPK may play a malevolent role (Mr Hyde!) to promote cancer*,* most likely by protecting transformed cells against stresses caused either when their growth rate outstrips the ability of their blood supply to deliver nutrients and oxygen or during periods of oxidative stress and/or DNA damage. In such scenarios, the presence of AMPK would increase the viability of the tumour cells and thereby potentially decrease survival of the patient, and in such cases it would be AMPK inhibitors rather than activators that might be therapeutically useful. The purpose of this review is to attempt to reconcile these two apparently conflicting roles of AMPK, and to discuss the different types of situation in which activators or inhibitors of the kinase might be efficacious.

## 2. AMPK—structure and regulation

AMPK appears to exist universally as heterotrimeric complexes comprising catalytic α subunits and regulatory β and γ subunits. Genes encoding these three subunits are found in the genomes of essentially all eukaryotes, suggesting that the AMPK system evolved very early during eukaryotic evolution [2]. In mammals, there are multiple genes encoding each subunit, generating two α (α1, α2), two β (β1, β2) and three γ subunits (γ1, γ2, γ3). These paralogues appear to have arisen during the two rounds of whole genome duplication that are thought to have occurred during the early development of the vertebrates [3]. The seven gene products (not counting splice and/or start-site variants) can form up to 12 αβγ combinations that display subtle differences in regulation and in tissue and subcellular distribution [3].

**Figure 1.**
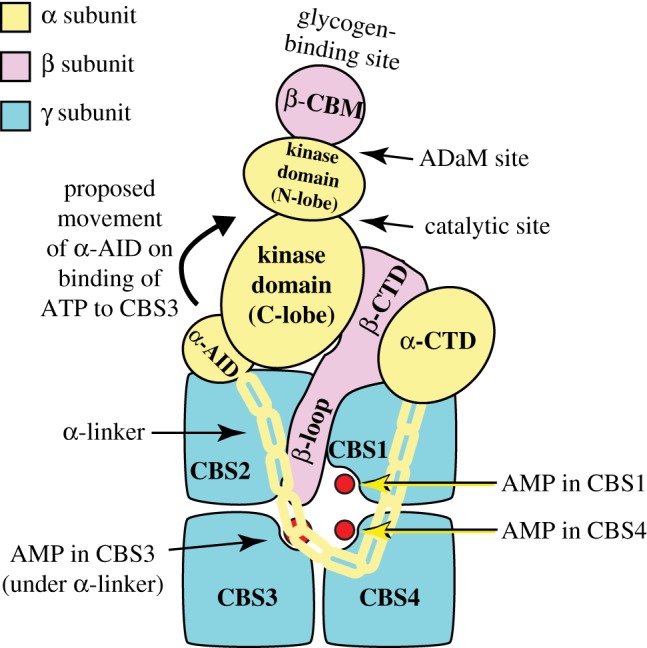
Schematic diagram of the structure of AMPK heterotrimers, with the different subunits colour coded (α, yellow; β, lilac; γ blue). Based on a structure of the human α1β2γ1 complex [19], although the structures of α2β1γ1 [17] and α1β1γ1 [18] complexes are very similar.

Crystal structures of three αβγ combinations from humans, i.e. α2β1γ1 [17], α1β1γ1 [18] and α1β2γ1 [19], as well as partial structures from mammals [20,21], budding yeast [22] and fission yeast [23,24], are now available. The generalized structure of a heterotrimeric AMPK complex is represented in a highly schematic form in figure 1. A current limitation of the existing structures of heterotrimeric complexes is that, in every case, the constructs were crystallized in active conformations, with the catalytic subunit phosphorylated at the activation site and allosteric activators bound at the regulatory sites. Due to the lack of structures in inactive conformations, we still only have a partial understanding of the conformational changes involved in the activation process.

### 2.1. Structure of the α subunits

Each AMPK-α subunit (coloured yellow in figure 1) has an N-terminal kinase domain with the small N-terminal lobe and larger C-terminal lobe typical of all members of the eukaryotic protein kinase (ePK) family, with the ATP-binding catalytic site in the cleft between the two lobes. Like many other ePKs, AMPK is only significantly active after phosphorylation within the so-called ‘activation loop’ of the C-lobe. In AMPK, the target for phosphorylation is a highly conserved threonine residue, which is conventionally referred to as Thr172 [25] although the exact residue numbering varies with species and isoform (in the view of figure 1, Thr172 is located on the far side of the C-lobe). In other ePKs, phosphorylation within the activation loop changes its conformation to reorient residues involved in both catalysis and protein substrate binding, thus greatly enhancing the reaction rate [26]. The principal upstream kinase phosphorylating Thr172 on AMPK was identified in 2003 to be a complex containing LKB1 and two accessory subunits, STRAD-α or -β and MO25-α or -β [14]. Binding of STRAD-α or -β (which are *pseudokinases*, structurally related to protein kinases but not active) is required for the kinase activity of LKB1, whereas MO25-α or -β appear to have a structural role to stabilize the complex [27]. The gene encoding LKB1 (called *STK11* in humans) had been previously identified as being involved in Peutz–Jeghers syndrome, a rare inherited cancer susceptibility; humans with this syndrome are almost always heterozygous for loss-of-function mutations in *STK11* [28]*.* Their major clinical problem is the development of frequent but benign intestinal polyps, which appear to be caused by haploinsufficiency in *STK11*. However, they also have a greatly increased risk of developing malignant cancers at multiple locations due to loss of heterozygosity in *STK11*, and often die at a relatively early age from such malignancies [28]. Loss-of-function mutations in *STK11* also frequently occur in many sporadic (i.e. non-inherited) cancers, especially in the commonest form of lung cancer, adenocarcinoma [3,29,30] (see also §6). LKB1 is therefore a classical tumour suppressor, and although its sequence showed that it was a member of the ePK family, the downstream target(s) that it phosphorylated were completely unknown until the finding that it phosphorylated and activated AMPK [14–16].

Following the kinase domain on each AMPK-α subunit (figure 1) is a compact bundle of three α-helices termed the *autoinhibitory domain* (*α*-*AID*) [18–20,24]. When ATP rather than AMP is bound at the regulatory site(s) on the γ subunit (see below), the α-AID is thought to interact with the kinase domain to clamp it in an inactive conformation [24]. The α-AID is linked to the globular C-terminal domain of the α subunit (α-CTD) by the *α-linker*, shown schematically as a yellow chain in figure 1. The α-linker is a region in an extended conformation that contains two conserved segments termed *α-regulatory interaction motifs* (*α*-*RIM1* and *α-RIM2*) [31]. These interact with the surface of the γ subunit containing the key regulatory adenine nucleotide-binding site (see §2.3), and movement of this linker is thought to transmit the effects of AMP or ADP binding from the regulatory γ subunit to the catalytic α subunit (see §2.4).

### 2.2. Structure of the β subunits

The β subunits (coloured lilac in figure 1) contain two conserved regions, the central carbohydrate-binding module (β-CBM) and the C-terminal domain (β-CTD), these being the only regions of the β subunits that are resolved in the current heterotrimer structures. The β-CBM causes a proportion of AMPK in mammalian cells to bind to glycogen particles [32,33]. One function of this may be to co-localize AMPK with glycogen synthase, the key enzyme of glycogen synthesis also found at the surface of the glycogen particle, both isoforms of which are phosphorylated and inactivated by AMPK [34,35]. The β-CBM, however, also has other functions (see §3.2 below). The β-CTD, on the other hand, plays a key structural role as the ‘core’ of the heterotrimeric complex, in that it cross-links the α-CTD and the γ subunit, via interactions that are highly conserved from fungi to mammals [21–23].

### 2.3. Structure of the γ subunits

The γ subunits (coloured blue in figure 1) are of particular interest because they contain the regulatory adenine nucleotide-binding sites. In all species, the γ subunits contain four tandem repeats of a sequence motif of around 60 amino acids known as a CBS repeat, so-named by Bateman [36] because they are also present in the enzyme *C*ystathione *β*-*S*ynthase and invariably occur as tandem repeats. CBS repeats have been identified in around 75 proteins in the human genome [37], and are also found in archaea and bacteria. Proteins containing them usually have just two tandem repeats, but the AMPK-γ subunits are unusual in having four. A single pair of repeats (known as a Bateman domain or module) forms a pseudodimer with a cleft between the repeats that (due to the approximate twofold symmetry) can provide two ligand-binding sites, although often only one is used. Bateman modules usually bind regulatory ligands containing adenosine (e.g. AMP, ATP, S-adenosyl methionine, NAD, diadenosine polyphosphate) or, less often, guanosine [37,38]. Consistent with this, the CBS repeats in the AMPK-γ subunits provide the critical binding sites for the regulatory nucleotides AMP, ADP and ATP [38]. The four CBS repeats in every AMPK-γ subunit form two Bateman modules that assemble ‘head to head’ to form a flattened disc with the adenine nucleotide-binding sites located close together in the centre, lining a narrow aqueous channel (figure 1) [17–19,21]. Given the presence of four repeats, it might have been expected that AMPK-γ subunits would bind four molecules of nucleotide, but all existing crystal structures suggest that they bind only three. These sites are now numbered according to which repeat in the linear sequence (CBS1 through CBS4) provides residues that bind the adenosine moiety of the nucleotide [39] (the phosphate groups may interact with residues from more than one repeat). Using this nomenclature, adenine nucleotides bind at CBS1, CBS3 and CBS4, while the CBS2 site appears to be always unoccupied. The CBS3 site is primarily accessible to solvent from one side of the disc of the γ subunit (facing the viewer in figure 1), and the CBS1 and CBS4 sites from the other.

### 2.4. Canonical regulation of AMPK by adenine nucleotides

AMP-activated protein kinase received its name [40] because it is allosterically activated by 5′-AMP [41]. When the assays are performed at physiologically relevant ATP concentrations (5 mM) allosteric activation can be as much as 10-fold [42]. However, even before LKB1 was identified as the upstream kinase and Thr172 as the phosphorylation site, it was realized that increases in the AMP : ATP ratio also promoted net phosphorylation of AMPK in intact cells [43]. This is now known to occur because AMP both enhances phosphorylation by LKB1 [44,45] and inhibits dephosphorylation by protein phosphatases [46]. Both effects are due to the binding of AMP to the *substrate*, AMPK, and not to the upstream kinase or phosphatase; indeed the LKB1 complex appears to have a constant activity in both energy-stressed and unstressed conditions [47]. To summarize, AMP binding has three effects on AMPK: (i) promoting Thr172 phosphorylation; (ii) inhibiting Thr172 dephosphorylation; (iii) triggering allosteric activation of kinase already phosphorylated on Thr172. These three mechanisms act synergistically and make the system respond to small increases in AMP in a very sensitive manner. It was subsequently reported that the effects of AMP binding on Thr172 phosphorylation [48] and dephosphorylation [20], although not on allosteric activation, could be mimicked by ADP, at least in cell-free assays. Our group has confirmed this, but found that the effect required concentrations of ADP up to 10-fold higher than those of AMP, at least for complexes containing γ1 and γ3 (γ2-containing complexes are more sensitive to ADP) [42,45]. Overall, we believe that increases in the AMP : ATP ratio remain the most important activating signal *in vivo*, although increases in the ADP : ATP ratio might make a secondary contribution.

How are these three effects of adenine nucleotide binding mediated by the three binding sites on the AMPK-γ subunits? Although it might seem tempting to propose that each effect is due to binding of nucleotides at one of the three sites, that simple model now seems to be untenable. Instead, all three effects appear to be due to binding of nucleotide at a single critical site, CBS3. The evidence supporting this may be briefly summarized as follows.

(1) CBS4 normally appears to contain a tightly bound ‘non-exchangeable’ molecule of AMP [21]. Similarly, although CBS1 can bind AMP in cell-free assays, it is estimated to have a 10-fold higher affinity for ATP than AMP. Since ATP is usually present in cells at up to 100-fold higher concentrations than AMP, this suggests that, in intact cells, CBS1 would always be occupied by ATP [49]. This leaves CBS3 as the site where ATP and AMP (or ADP) could exchange with each other.
(2) The R531G mutation in the AMPK-γ2 subunit, one of up to 14 mutations that cause an inherited heart disease [50], completely blocks both allosteric activation and increased net Thr172 phosphorylation by AMP [38,51]. Although it is actually located in CBS4, the positively charged side chain of Arg531 interacts with the α-phosphate of AMP bound in CBS3 [21,49] (note that in [21] the CBS3 site was referred to as site 1; see [39] for revised nomenclature of binding sites).

If CBS3 is the critical site for all three effects of AMP, what are the functions of nucleotide binding at CBS1 and CBS4? All three sites are located very close together in the centre of the γ subunit, where they are likely to interact with each other. Gu *et al.* [49] have provided evidence that binding of ATP at CBS1 alters the conformation of the neighbouring CBS4 site such that the latter binds only AMP in a non-exchangeable manner. They further propose that binding of AMP at CBS4 then enhances the affinity of AMP relative to ATP at CBS3. In particular, binding of AMP at CBS4 repositions the side chain of Arg531 such that it provides an additional positive charge to bind the two negatively charged oxygen atoms on the α-phosphate of AMP in CBS3 (note that the α-phosphates of ADP and ATP, unlike that of AMP, carry only single negative charges) [49]. If this is correct, constitutive binding of ATP to CBS1 and AMP to CBS4 effectively ‘tunes’ the affinity of the CBS3 site for the different nucleotides that can bind there. This model explains how AMPK achieves the difficult task of sensing changes in AMP in the presence of much higher concentrations of ATP and ADP. An additional explanation for the preference of the CBS3 site for AMP over ATP is that all three sites on the AMPK-γ subunits appear to preferentially bind free ATP^4−^ rather than the Mg.ATP^2−^ complex [21,23,38,49], although only around 10% of ATP in cells is thought to be present in the Mg^2+^-free form.

How is the effect of displacement of ATP by AMP at CBS3 transmitted to the catalytic (α) subunit? Consistent with the idea that CBS3 is the critical site for activation, the heterotrimer structures show that, when AMP is bound at CBS3, the α-linker binds to that face of the γ subunit, with α-RIM1 binding across the unoccupied CBS2 site and α-RIM2 physically contacting AMP bound at CBS3 (figure 1). Although there are no crystal structures to confirm this, other biophysical approaches suggest that, when ATP displaces AMP at CBS3, the α-linker dissociates from the surface of the γ subunit containing the CBS3 site [19,52]. This is thought to release the α-AID to rotate back into its inhibitory position behind the kinase domain, with this being prevented when AMP is bound at CBS3 by the interaction of the α-linker with the CBS3 site. Consistent with this model, mutations that would affect the interactions between α-RIM1/α-RIM2 and the γ subunit abolish allosteric activation by AMP [31].

While this model nicely accounts for allosteric activation by AMP, the accompanying conformational changes may also alter the exposure of Thr172 for phosphorylation and dephosphorylation, although that aspect is currently less well understood. It also remains unclear why ADP binding has effects on Thr172 phosphorylation despite the fact that, unlike AMP, it does not cause allosteric activation. Finally, as well as the ‘canonical’ activation by changes in adenine nucleotide ratios discussed above, AMPK can also be activated by several non-canonical mechanisms that will now be briefly described.

### 2.5. Non-canonical activation by increases in intracellular Ca^2+^, by glucose deprivation and by DNA damage

As well as LKB1, Thr172 can also be phosphorylated by the Ca^2+^/calmodulin-dependent protein kinase CaMKK2 [7–9], which means that AMPK can be activated by increases in intracellular Ca^2+^ ions even in the absence of any changes in adenine nucleotide ratios. This occurs, for example, in response to hormones and agonists sensed by G protein-coupled receptors that are coupled via G_q_/G_11_ to release inositol-1,4,5-trisphosphate (IP_3_) from the plasma membrane, which in turn triggers release of Ca^2+^ from the endoplasmic reticulum. Such agonists include, in endothelial cells, thrombin acting at protease-activated receptors and vascular endothelial cell growth factor acting at VEGF receptors [53,54] as well as, in specific neurons of the hypothalamus, ghrelin acting at GHSR1 receptors [55]. The latter effect is important in promotion of appetite during fasting [5], and the role of CaMKK2 in this pathway can explain previous findings that CaMKK2 inhibitors depress appetite in wild-type mice, although not in CaMKK2 knockouts [56].

It has been known for many years that glucose deprivation of mammalian cells activates AMPK [57], and this treatment is often used to switch on AMPK in cultured cells. In fact, genes encoding the budding yeast orthologue of AMPK (the SNF1 complex) were originally identified via mutations that prevented the normal changes in gene expression in response to glucose deprivation [58]. For many years, it was assumed that glucose deprivation activated AMPK by interfering with catabolic ATP production, and thus activated AMPK via the canonical, AMP-dependent mechanism (§2.4). This does indeed seem to be the case in some established tumour cell lines, perhaps because they are highly glycolytic and have a high dependency on glucose for ATP production. However, in other cells such as immortalized mouse embryo fibroblasts (MEFs) it has been found that glucose deprivation activates AMPK without changing AMP : ATP or ADP : ATP ratios, as long as an alternative carbon source such as glutamine is available; similar AMP/ADP-independent activation is also observed in rat liver during starvation *in vivo* [10]. In such cases, activation is thought to occur via a complex mechanism involving the direct sensing of the glycolytic intermediate fructose-1,6-bisphosphate (FBP) by FBP aldolase, and the recruitment of AMPK to a ‘super-complex’ on the lysosomal membrane involving the vacuolar-ATPase, the Ragulator complex, Axin, LKB1 and AMPK. Although this mechanism may operate in tumour cells that are dependent on rapid glucose uptake, a full discussion of it is beyond the scope of this article and interested readers are referred to the original papers [10,59,60] or a recent review [2].

A third type of non-canonical activation of AMPK occurs in response to DNA damage. This was originally reported to occur in response to the topoisomerase II inhibitor etoposide [12], and was later observed following treatment of cells with ionizing radiation [13]. Both treatments cause double-strand breaks in DNA, and are often used in cancer treatment. Double-strand DNA breaks are known to be detected by ATM, a member of the phosphatidylinositol 3-kinase-like kinase (PIKK) family, and the effects of etoposide to activate AMPK were originally claimed to be dependent on ATM, because the effects appeared to be reduced in ATM-deficient cells [12]. In addition, ATM is known to phosphorylate LKB1 at Thr366 [61], and it was reported that AMPK activation by etoposide in cells was reduced by siRNA-mediated knockdown of either ATM or LKB1 [12,62], suggesting the existence of a kinase cascade from ATM to LKB1 to AMPK. However, this cannot be the primary mechanism, because both etoposide [12] and ionizing radiation [13] still activate AMPK in LKB1-null tumour cells. Moreover, our laboratory showed that AMPK activation by etoposide was not blocked by the ATM inhibitor KU-55993, despite the fact that the inhibitor did block phosphorylation of known ATM substrates [11]. We went on to show that AMPK activation by etoposide in LKB1-null cells was mediated by Thr172 phosphorylation catalysed by CaMKK2, and that this was associated with increases in Ca^2+^ within the nucleus. Interestingly, only AMPK complexes within the nucleus containing the α1 isoform were activated, even though α2 was also expressed in the cells under study. Perhaps most interesting of all, activating AMPK in LKB1-null cells (using the Ca^2+^ ionophore A23187 to activate CaMKK2) provided significant protection against cell death induced by etoposide. The most likely mechanism to explain this was that A23187 caused a G1 cell cycle arrest, thus restricting entry of cells into S phase where they are particularly susceptible to DNA damage. This hypothesis was supported by the fact that the G1 cyclin-dependent kinase inhibitor palbociclib caused a very similar degree of protection against cell death in those cells where it caused G1 arrest, but not in those where it did not [11]. These results are significant, because they suggest that genotoxic treatments such as etoposide and ionizing radiation might be more effective for cancer treatment if they were combined with inhibitors that prevent AMPK activation, and the consequent protection that AMPK can provide against genotoxic stress. This point is addressed further in §6 below.

## 3. Pharmacological activation and inhibition of AMPK

**Figure 2.**
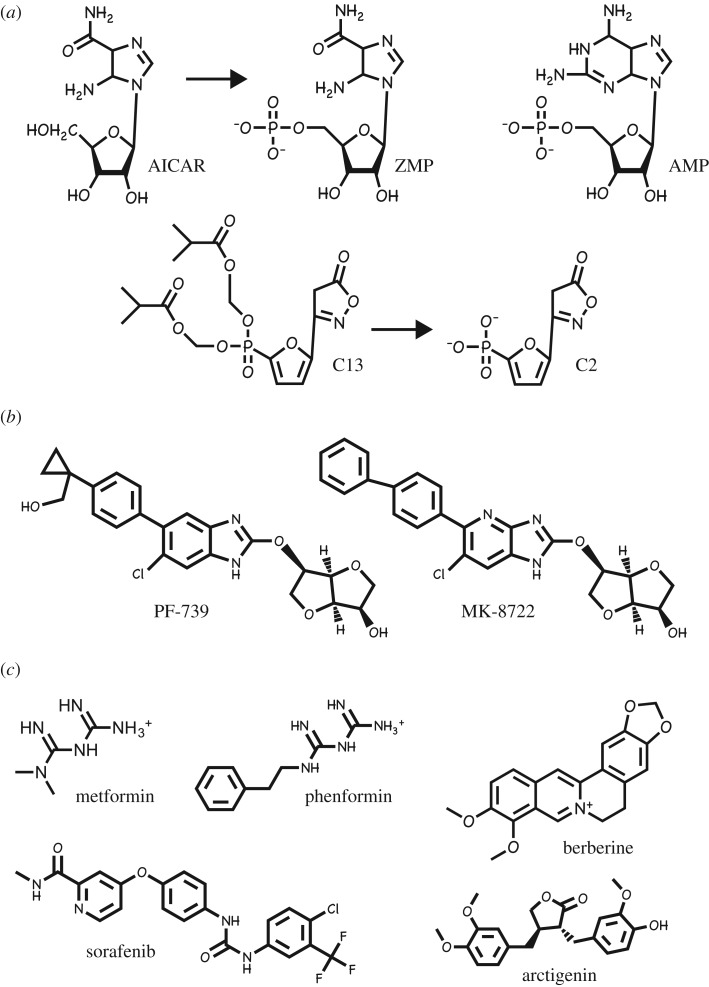
Structures of a number of AMPK activators. They have been classified according to their mechanisms of activation of AMPK (see §§3.1–3.3). (*a*) Pro-drugs that are converted inside cells to AMP analogues. (*b*) Compounds that bind in the allosteric drug and metabolite (ADaM) site. (*c*) Compounds that activate indirectly by inhibiting mitochondrial ATP synthesis.

The realization that AMPK acts as a metabolic master switch, which transforms cellular metabolism from an anabolic to a catabolic state, originally suggested that activators of AMPK might be useful in treating disorders of energy balance such as obesity and type 2 diabetes [63]. Similarly, the discoveries that AMPK inhibited both cell growth and cell proliferation suggested that activators might also be useful in the treatment of cancer [64]. Over the past 20 years, scores of compounds that pharmacologically activate AMPK have been described, a few of which are shown in figure 2. These are discussed in §§3.1–3.3 according to their likely modes of action. There has been much less emphasis on the development of inhibitors, but these are briefly discussed in §3.4.

### 3.1. Pro-drugs that are converted inside cells to AMP analogues

The first compound shown to activate AMPK in intact cells was the adenosine analogue 5-aminoimidazole-4-carboxamide riboside (AICAR), which is taken up into cells via adenosine transporters [65] and converted by adenosine kinase into the equivalent monophosphorylated nucleotide, ZMP [66–68] (figure 2*a*). ZMP mimics all three of the effects of AMP described in §2.4 [66], and AICAR has been much used as an experimental tool to activate AMPK in intact cells and *in vivo*. It should be noted, however, that ZMP is much less potent as an AMPK activator than AMP, and AICAR only activates AMPK in intact cells because intracellular ZMP accumulates to millimolar concentrations, even higher than the external concentrations of AICAR [66]. The use of AICAR is no longer recommended by the present authors, because ZMP has known off-target effects (e.g. it also mimics the effects of AMP to activate skeletal muscle glycogen phosphorylase [69] and inhibit hepatic fructose-1,6-bisphosphatase [67,70]), and because much more specific activators are now available. One such is C13, a derivative of another adenosine analogue termed C2 that has been esterified on two oxygen atoms of its phosphonate group to make it more cell permeable [71]. C13 is indeed readily taken up by cells, but is then converted into C2 by cellular esterases (figure 2*a*). Remarkably, C2 is an even more potent activator of AMPK than AMP itself, although it should be noted that it is specific for AMPK complexes containing the α1 isoform, and is inactive on α2 complexes [72]. The high affinity of C2 may arise because it binds, unexpectedly, to the AMPK-γ subunits in a somewhat different orientation than AMP [73].

### 3.2. Compounds that bind in the allosteric drug and metabolite (ADaM) site

In the structures of AMPK heterotrimers containing either β1 [17,18] or β2 [19], the β-CBM interacts with the N-lobe of the kinase domain of the α subunit via the surface opposite to its glycogen-binding site (figure 1). The cleft between these domains forms the binding site for novel ligands acting on AMPK, which in most cases came out of high-throughput screens that searched libraries of synthetic chemicals for allosteric activators of AMPK. The first to be discovered was the thienopyridone A-769662 [74] but at least 10 have now been reported, including PF-739 [75] and MK-8722 [76] (figure 2*b*). All activate β1 complexes with higher potency than β2 complexes, and this makes some of them (including A-769662 [77]) highly selective for the former. As well as causing allosteric activation, binding of these compounds also inhibits Thr172 dephosphorylation in cell-free assays [78,79], although in intact cells the predominant effect appears to be allosteric, since large changes in phosphorylation of the AMPK target acetyl-CoA carboxylase in response to these agonists are usually only accompanied by modest changes in Thr172 phosphorylation [78].

One of the curious features of the ligands currently known to bind at this site is that almost all of them are synthetic chemicals rather than natural products. However, many in the field believe that these compounds may be mimicking the effect of some natural metabolite that binds to this site, which is why it has been termed the ‘allosteric drug and metabolite’ (ADaM) site [80]. The only natural product currently known to bind to this site is salicylate, a compound made by plants that acts as a hormone signalling infection by pathogens [81]. In the form of extracts of willow bark, salicylates have been used by humans as medicines since ancient times, and they are still in very wide use as the synthetic derivative acetyl salicylic acid (ASA or aspirin), which is rapidly broken down to salicylate once it enters the circulation. Although aspirin itself is a potent irreversible inhibitor of the cyclo-oxygenases involved in biosynthesis of prostanoids such as thromboxanes [82], salicylate activates AMPK by direct binding at the ADaM site, which occurs at concentrations reached in plasma of patients taking high doses of aspirin and other salicylate-based drugs [81]. Interestingly, regular use of aspirin, usually taken to reduce the formation of blood clots via inhibition of thromboxane synthesis, is associated with a reduced incidence of cancer [83]. Whether this can be explained entirely by inhibition of cyclo-oxygenases, or whether it involves some other target such as AMPK, currently remains unclear.

### 3.3. Compounds, including biguanides, that activate AMPK indirectly by inhibiting mitochondrial ATP synthesis

Metformin and phenformin (figure 2*c*) are synthetic biguanides derived from *galegine* (isoprenyl guanidine) [84], a natural product from the plant goat's rue or *Galega officinalis*, which was well known as a herbal remedy in seventeenth century England [85]. Both biguanides were introduced for treatment of type 2 diabetes in the 1950s, although phenformin was withdrawn in most countries in the 1970s because its use was associated with the rare but life-threatening side effect of lactic acidosis. The risk of lactic acidosis is much lower with metformin, which has subsequently become the drug of first choice in the treatment of type 2 diabetes worldwide. Although biguanides have been used since the 1950s, the first clues to their mechanism of action did not emerge until 2000, when they were reported to inhibit complex I of the mitochondrial respiratory chain, thus explaining the risk of lactic acid accumulation [86,87]; they have subsequently also been shown to inhibit the mitochondrial ATP synthase [88]. Clearly, inhibition of mitochondrial ATP synthesis would be expected to increase cellular ADP : ATP and AMP : ATP ratios and thus activate AMPK by the canonical mechanism. Indeed, activation of AMPK by biguanides in intact cells and *in vivo* was reported in 2001 [89], and it was subsequently confirmed that this was caused by increases in AMP and/or ADP [51], although metformin may also activate AMPK via the non-canonical lysosomal pathway [90]. Metformin has two major clinical effects: (i) inhibiting glucose production by the liver and (ii) enhancing insulin sensitivity of tissues such as liver and skeletal muscle. Surprisingly, studies with liver-specific double AMPK (α1^−/−^ α2^−/−^) knockout mice showed that the rapid effects of metformin on liver glucose production were AMPK independent, despite the fact that they were accompanied by increases in cellular AMP : ATP ratios [91]. These acute effects of metformin now appear to be due to direct allosteric inhibition of the gluconeogenic enzyme fructose-1,6-bisphosphatase by AMP [70]. Despite this, studies of mice with double knock-in mutations of the single serine residues that are targeted by AMPK in ACC1 (S79A) and ACC2 (S212A) suggested that the longer term insulin-sensitizing effects of metformin are indeed mediated by AMPK [92]. These mice, in which AMPK no longer acutely inhibits fatty acid synthesis or activates fatty acid oxidation, accumulate excess di- and tri-glycerides in liver and muscle, which is accompanied by insulin resistance. Although wild-type mice developed a similar degree of insulin resistance when placed on a high-fat diet, insulin sensitivity in the knock-in mice did not deteriorate further, possibly because they were already synthesizing so much fat. However, when the high-fat-fed mice were treated with metformin for six weeks, this reversed the insulin resistance of the wild-type mice but had no effect in the knock-in mice. Thus, the longer term effects of metformin on insulin sensitivity, although not its short-term effects on hepatic glucose production, are due to modulation of lipid metabolism by AMPK, most likely by reducing the excessive storage of lipids in tissues such as liver and skeletal muscle [92].

Following the initial findings that AMPK was activated by biguanides [89], and that the tumour suppressor LKB1 acted upstream of AMPK [14], the question of whether biguanide use had any influence on cancer was addressed. Retrospective studies suggested that the use of metformin in patients with type 2 diabetes in the Tayside region of Scotland was associated with a significant (around 30%) reduction in the incidence of cancer [93]. This association has since been confirmed in studies of many other diabetic cohorts [94–96], although its validity has been challenged due to the possibility of time-related biases [97] and it remains just a correlation, with no proof of direct causation. In addition, even if the association is valid, it does not necessarily imply that metformin acts directly on AMPK within the tumours themselves, rather than indirectly via AMPK-dependent or -independent effects on other tissues or organs. For example, since metformin is currently only used to treat type 2 diabetes, we do not know whether its use would be associated with reduced cancer incidence in subjects without diabetes (although there have been small trials in patients with breast or endometrial cancer, these were only ‘window-of-opportunity’ trials to assess various markers in the short period prior to surgery [98–101]). Note also that the different cancer incidence in patients with type 2 diabetes taking metformin is observed when comparing with those on other medications [93–96]. Metformin enhances insulin sensitivity and thus reduces insulin release, but many of the other commonly used medications, such as sulfonylureas and glucagon-like peptide-1 agonists, work in part by enhancing insulin secretion, while some subjects are even treated directly with insulin. Insulin is, of course, a growth factor that promotes proliferation of cells by activating the Akt pathway. One explanation of the apparent protective effect of metformin against cancer in patients with diabetes is therefore that, unlike most other treatments, it *reduces* rather than increases the levels of insulin, with high insulin levels being responsible for *increased* cancer incidence in patients on other medications [102]. Indeed, a related phenomenon is seen in patients with cancer who are treated with phosphatidyl-inositol 3-kinase (PI3 K) inhibitors, who often secrete extra insulin to compensate for the insulin resistance induced by the drugs, thus reducing their anti-cancer efficacy. Experiments with mouse models suggest that this effect can be overcome by additional dietary or pharmacological treatments that reverse the insulin resistance induced by these drugs [103].

There are many other compounds that activate AMPK by inhibiting mitochondrial ATP synthesis, one example being resveratrol [51], which inhibits the mitochondrial ATP synthase [104]. Another is sorafenib, originally developed as an inhibitor of receptor-linked tyrosine kinases such as the VEGF and platelet-derived growth factor (PDGF) receptors and used to treat some liver, kidney and thyroid cancers [105]. However, it also activates AMPK at therapeutically relevant concentrations by inhibiting the respiratory chain [106]. Remarkably, more than 100 natural products derived from traditional Asian medicines have within the last few years also been shown to activate AMPK in intact cells [107], and the effects of at least two of them, i.e. berberine [51] and arctigenin [108], appear to be due to inhibition of complex I of the mitochondrial respiratory chain. We suspect that many of the others may also work through inhibition of either complex I or the ATP synthase, which are both large, membrane-bound complexes containing no less than 44 and 14 protein subunits, respectively. It is perhaps not surprising that many hydrophobic compounds might find inhibitory binding sites within these complexes. This class of AMPK activator is particularly diverse in structure (e.g. those in figure 2*c*), indicating that they may interact with distinct sites. Many of the natural products that activate AMPK may be produced by plants to provide a chemical defence to deter grazing by insects or other animals, or infection by pathogens, and poisoning of complex I or the ATP synthase would seem to represent good ways to achieve those aims. Interestingly, many of these toxic plant products are stored within the plants that synthesize them either in the vacuole or in the cell wall [109], where they would not come into contact with the plant's own mitochondria.

### 3.4. AMPK inhibitors

At present, no specific AMPK inhibitors are available. The only AMPK inhibitor that has been widely used in the literature is *compound C* (also known as *dorsomorphin*). Although developed as an AMPK inhibitor, the claim that it was specific for AMPK came from the original report that it did not inhibit a panel of just five other protein kinases [89]. However, in a screen of 70 protein kinases, nine were inhibited to a greater extent than AMPK [110], while in a more recent screen of 120 kinases documented in the MRC Kinase Inhibitor Database (www.kinase-screen.mrc.ac.uk/kinase-inhibitors) no less than 30 were inhibited to a greater extent than AMPK. The use of compound C cannot therefore be recommended, even as an experimental tool. Other AMPK inhibitors have been reported [111,112], but have not yet been widely used.

## 4. Downstream targets of AMPK

Once activated, AMPK phosphorylates numerous downstream proteins, with at least 60 being identified as well-established targets in a recent review [113]. The core recognition motif for AMPK is well defined: it requires a basic residue (R, K or H) either three or four residues N-terminal to the phosphorylated serine/threonine (referred to as the P-3 and P-4 positions) as well as hydrophobic residues (L, M, I, F or V) at P-5 and P+4 [113–115]. The ACC1 isoform of acetyl-CoA carboxylase, which is a particularly good substrate for AMPK, has additional specificity determinants N-terminal to this core motif, which are not present in all downstream targets. These are another basic residue at P-6, and an amphipathic α-helix running from P-5 to P-16 that binds in a hydrophobic groove on the surface of the C-lobe of the AMPK kinase domain [116]. We discuss some of these targets below, focusing on those that may be particularly relevant to the role of AMPK in cancer.

### 4.1. Proteins and genes involved in catabolic pathways

**Figure 3.**
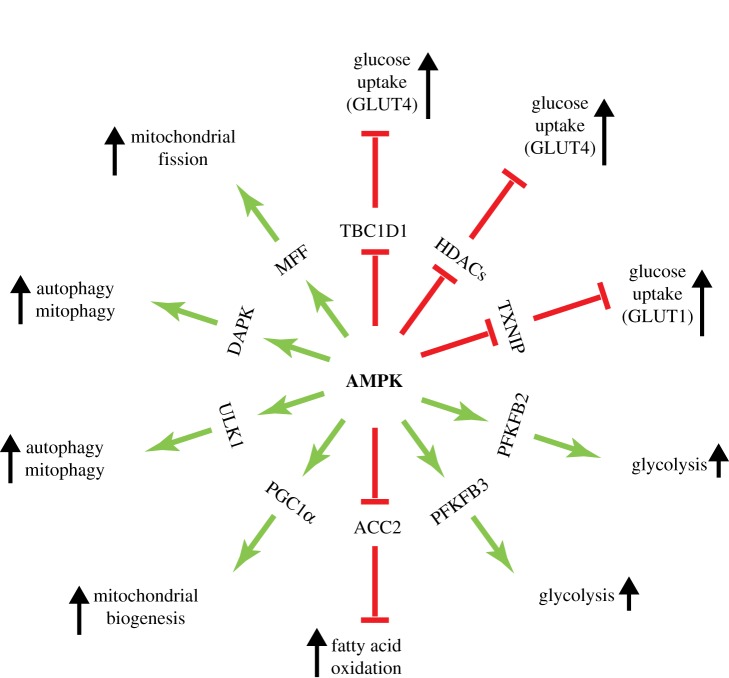
A ‘wheel’ of downstream targets and the pathways they regulate, focusing on catabolic processes that are activated by AMPK.

Catabolic processes switched on by AMPK are summarized in figure 3. In many cell types, depending on the expression of specific glucose transporters (GLUTs), AMPK activation enhances glucose uptake. In skeletal muscle, AMPK acutely promotes translocation of vesicles containing GLUT4 from intracellular vesicles to the plasma membrane, in part by a mechanism involving phosphorylation of the Rab-GAP protein TBC1D1 [117]. In the longer term, AMPK also increases expression of GLUT4 protein via a mechanism that may involve direct phosphorylation of class IIa histone deacetylases (e.g. HDAC5) [118], which appears to cause their exclusion from the nucleus [119] and therefore promotes net acetylation and transcriptional activation at the GLUT4 promoter. AMPK activation also acutely activates glucose transport by the more widely expressed glucose transporter GLUT1 [120], in part via phosphorylation and consequent degradation of TXNIP, an α-arrestin family member that appears to promote internalization of GLUT1 as well as reduced levels of its mRNA [121]. In some but not all cells, AMPK acutely stimulates glycolytic flux via a mechanism involving direct phosphorylation of 6-phosphofructo-2-kinase/fructose-2,6- bisphosphatase, the enzyme that makes and breaks down fructose-2,6-bisphosphate via distinct domains of a bienzyme polypeptide [122]. Phosphorylation by AMPK increases the kinase activity, thus increasing the cellular concentration of fructose-2,6-bisphosphate, a potent allosteric activator of the glycolytic enzyme 6-phosphofructo-1-kinase [122]. However, this mechanism is limited to specific cell types, because only the PFKFB2 [123] and PFKFB3 [124] isoforms are targets for AMPK. PFKFB2 is expressed in cardiac myocytes and some other tissues, while alternative splicing or differential promoter usage yields two main isoforms of PFKFB3 that differ by a short C-terminal sequence; these are the so-called ubiquitous (or constitutive) isoform and the inducible isoform [122]. The expression of the inducible form is very low in most adult tissues, but is increased by pro-inflammatory stimuli in monocytes and macrophages [124] and it is constitutively expressed in many tumour cells [125].

Although AMPK can therefore acutely activate ATP production by glycolysis in some cell types, in the longer term it tends to promote mitochondrial oxidative metabolism instead, which is much more efficient in terms of ATP production per glucose consumed (≈36 ATP per glucose by oxidative metabolism, as opposed to only two by glycolysis). Oxidative metabolism is, however, less compatible with providing precursors for cell growth, so it tends to be used to a greater extent in quiescent rather than proliferating cells [126]. In the short term, AMPK activates the uptake of fatty acids into mitochondria via phosphorylation of the acetyl-CoA carboxylase isoform ACC2 [127]. While ACC1, the first AMPK target to be identified, is thought to produce the cytoplasmic malonyl-CoA used in fatty acid synthesis, ACC2 localizes to mitochondria [128] and is thought to produce the mitochondrial malonyl-CoA that inhibits uptake of fatty acids into mitochondria via the carnitine:palmitoyl transferase system. Phosphorylation of ACC2 lowers malonyl-CoA and therefore relieves inhibition of carnitine:palmitoyl-CoA transferase-1 (CPT1), thus causing acute promotion of mitochondrial fatty acid oxidation [127].

In the longer term, AMPK activation has several effects on mitochondria that enhance their capacity to produce ATP at a rapid rate. Firstly, AMPK activation promotes mitochondrial biogenesis itself, involving increased replication of mitochondrial DNA as well as expression of many nuclear-encoded mitochondrial proteins, by activating the transcriptional co-activator PGC-1α [129]. This is effected either by direct phosphorylation of PGC-1α [130] or by increasing the cellular concentration of NAD^+^, a cofactor required for deacetylation and activation of PGC-1α by SIRT1 [131]. Secondly, being the major site of cellular production of reactive oxygen species, mitochondrial components are particularly prone to oxidative damage, and if this affects their function mitochondria need to be removed and their contents recycled by the targeted form of autophagy known as mitophagy. Relevant to this, AMPK has been shown to promote both autophagy and mitophagy either by phosphorylation of the protein kinase that triggers autophagy, ULK1 [132,133], or by phosphorylation of the Ca^2+^/calmodulin-dependent kinase DAPK, generating a Ca^2+^/calmodulin-independent form that phosphorylates the key autophagy protein Beclin-1 [134]. Thirdly, mitochondria are now known to exist, especially in quiescent cells, not as small separate organelles, but as branching networks of tubules that can be almost as long as the cell containing them [135]. If any regions of such a network become damaged, they need to be segregated off from healthy regions via the process of *mitochondrial fission*, so that they become small enough to be recycled by mitophagy. Intriguingly, AMPK activation has been shown to promote mitochondrial fission by direct phosphorylation of *mitochondrial fission factor* (*MFF*) [136]. These findings are consistent with one aspect of the phenotype of skeletal muscle-specific double AMPK knockouts (either α1 and α2 [137] or β1 and β2 [138]), in which muscle accumulates abnormally shaped and apparently malfunctioning mitochondria. Overall, AMPK appears to play several crucial roles in mitochondrial homeostasis. Since mitochondria are the main source of cellular ATP in most cells, this makes perfect sense for a signalling pathway that is activated by energy stress and/or glucose deprivation.

### 4.2. Proteins and genes involved in anabolic pathways

**Figure 4.**
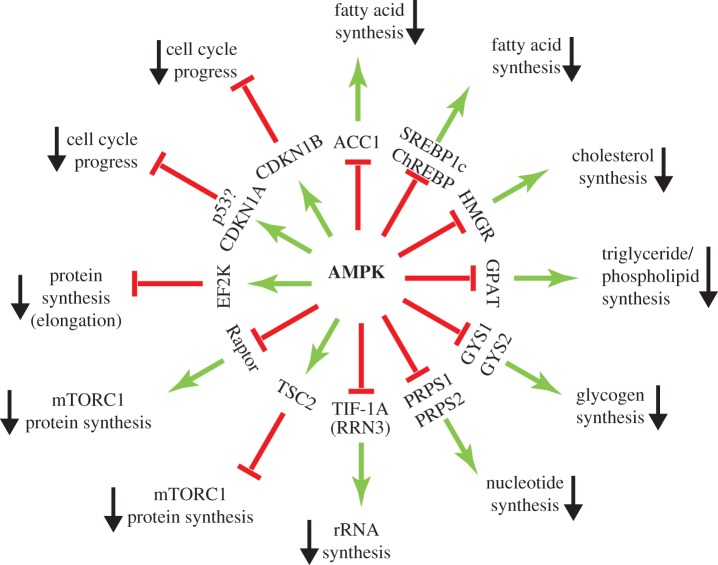
A ‘wheel’ of downstream targets and the pathways they regulate, focusing on anabolic and other processes that are inhibited by AMPK.

As well as switching on catabolic pathways that generate ATP, AMPK also switches off almost all major anabolic pathways (figure 4). AMPK was originally defined via its ability to phosphorylate and inactivate both acetyl-CoA carboxylase (ACC1) and 3-hydroxy-3-methylglutaryl-CoA reductase (HMGR), the key regulatory enzymes of fatty acid and sterol synthesis, respectively [139,140]. Fatty acid synthesis is a significant energy-consuming pathway in rapidly dividing tumour cells, being a major consumer of both ATP (consumed in the ACC1 reaction) and NADPH (consumed in the two reductive steps catalysed by the fatty acid synthase complex). Indeed, ACC1 remains one of most rapidly phosphorylated substrates for AMPK, and the phosphorylation of Ser80 (human numbering) on ACC1, monitored using a phosphospecific antibody, remains the most reliable and widely used cellular marker of AMPK function.

As well as acutely inhibiting fatty acid synthesis by direct phosphorylation of ACC1 (which catalyses the first two steps of fatty acid synthesis from acetyl-CoA), AMPK activation also downregulates expression of the genes encoding ACC1 (*ACACA*) as well as the gene (*FASN*) encoding the fatty acid synthase complex, a dimeric multienzyme polypeptide that catalyses the remaining seven reactions leading to a saturated C16 fatty acid (palmitate). The AMPK targets responsible for these effects may be the transcription factors sterol response element binding protein-1c (SREBP1c) [141] and/or the carbohydrate response element binding protein (ChREBP) [142], which have both been reported to be directly phosphorylated by AMPK.

As well as inhibiting *de novo* synthesis of fatty acids, AMPK inhibits synthesis of triglyceride and phospholipid synthesis by inactivating the first enzyme (*glycerol phosphate acyl transferase, GPAT*) involved in the synthesis of the common intermediate diacylglycerol [143], although whether this is due to direct phosphorylation of the enzyme remains unclear. Two direct targets for AMPK are the muscle (*GYS1*) [34] and liver (*GYS2*) [35] isoforms of glycogen synthase, which catalyse the transfer of glucose from UDP-glucose to the growing non-reducing ends of the glycogen particle. Both are inactivated by phosphorylation at equivalent N-terminal sites by AMPK, although this inactivation is over-ridden by high concentrations of the feed-forward allosteric activator of glycogen synthase, glucose-6-phosphate [144]. The need to co-localize AMPK with glycogen synthase may be one reason why AMPK-β subunit isoforms in all species carry a carbohydrate-binding module (β-CBM; see figure 1).

Another key pathway in growing cells is biosynthesis of nucleotides. The ribose or deoxyribose components of nucleotides are derived from ribose-5-phosphate generated in the pentose phosphate pathway. It has recently been reported that PRPS1 and PRPS2, the two major isoforms of phosphoribosyl pyrophosphate synthetase that metabolize ribose-5-phosphate in the first step of nucleotide biosynthesis, are phosphorylated and inactivated by direct phosphorylation by AMPK [145]. The pentose phosphate pathway also generates NADPH that is used for fatty acid biosynthesis, as well as for regenerating reduced glutathione used to combat oxidative stress. The large requirement for NADPH and nucleotide biosynthesis in rapidly proliferating cells may be one reason why they exhibit rapid glucose uptake to provide input of glucose into the pentose phosphate pathway.

Nucleotides are, of course, the building blocks for RNA and DNA. In rapidly proliferating thymocytes, the addition of actinomycin D (a general inhibitor of RNA synthesis) reduces oxygen uptake by as much as 15% [146], suggesting that RNA synthesis accounts for at least that percentage of total ATP turnover. Since up to 80% of the total RNA in a typical cell is ribosomal RNA (rRNA), synthesis of the latter is a major anabolic pathway and consumer of energy in proliferating cells. Consistent with this, AMPK activation has been found to inhibit rRNA synthesis by direct phosphorylation of the transcription factor for RNA polymerase I, TIF-1A (encoded by the *RRN3* gene) [147].

Arguably the most important biosynthetic pathway in proliferating cells is translation (protein synthesis). Over 50% of the dry weight of most cells is protein while, in the proliferating thymocyte system mentioned above, inhibition of protein synthesis reduced oxygen uptake by more than 20% [146]. AMPK switches off translation by at least two mechanisms. Firstly, it inactivates the target of rapamycin complex-1 (TORC1), which is known to promote the initiation step of ribosomal protein synthesis by triggering the phosphorylation of multiple proteins, including eukaryotic initiation factor-4E binding protein-1 (EIF4EBP1) and ribosomal protein S6 kinase-1 (RPS6KB1) [148]. Phosphorylation of EIF4EBP1 leads to selective translation of mRNAs containing 5′-terminal oligopyrimidine (5′-TOP) sequences, which often encode mRNAs encoding proteins required for rapid cell growth, including most ribosomal proteins as well as other proteins involved in translation [149]. AMPK inactivates mTORC1 by at least two mechanisms, i.e. inhibitory phosphorylation of the Raptor subunit that targets the complex to downstream targets and to the lysosome where it is activated, and activatory phosphorylation of TSC2, which forms a key part of the TSC1:TSC2:TBC1D7 complex. The latter has a *Rheb:GTPase activator protein* (*Rheb:GAP*) domain on TSC2 that converts the mTORC1-activating G protein Rheb to its inactive GDP-bound form [150]. Secondly, AMPK inhibits the elongation step of ribosomal protein synthesis by promoting phosphorylation of elongation factor-2 at Thr56. This residue is phosphorylated not by AMPK itself but by elongation factor-2 kinase (EF2 K), a member of the atypical protein kinase (aPK) family that is activated by Ca^2+^/calmodulin. AMPK appears to activate EF2 K in part by direct phosphorylation [151] and in part by inactivating mTORC1, with EF2 K being phosphorylated and inactivated by p70S6K1 downstream of mTORC1 [152,153].

### 4.3. Progress through the cell cycle

As well as inhibiting most major biosynthetic pathways, AMPK activation can also cause cell cycle arrest (figure 4). As long ago as 2001, it was reported that the AMPK activator 5-aminoimidazole-4-carboxamide riboside (AICAR) caused arrest in the G1 phase of the cell cycle in HepG2 cells, which was attributed to phosphorylation of the transcription factor p53 at Ser15, and consequent increased expression of the G1 cyclin-dependent kinase inhibitor p21^CIP1^ (CDKN1A) [154]. This was followed by a demonstration that both AICAR and low glucose caused cell cycle arrest in MEFs [155]. These effects were at least partially dependent upon p53, because they were reduced in p53^−/−^ MEFs. Although both AICAR and glucose deprivation can have off-target, AMPK-independent effects, the effects of low glucose also appeared to be AMPK dependent because they were reduced by expression of a dominant negative AMPK mutant (a kinase-inactive AMPK-α mutant that inhibits endogenous AMPK-α subunits by competing for available β and γ subunits). This group also reported that an activated kinase domain construct of AMPK could directly phosphorylate p53 at Ser15 [155]. However, the sequence around Ser15 is not a good fit to the AMPK recognition motif, lacking a basic residue at P-4 or P-3 (in fact, with an acidic residue at P-4 instead). It seems more likely that the phosphorylation of p53 observed in response to AMPK activation is indirect.

Another potential mechanism by which AMPK causes G1 arrest involves phosphorylation of another cyclin-dependent kinase inhibitor, p27^WAF1^ (CDKN1B). In breast cancer cells (MCF-7), p27 was found to be phosphorylated at its C-terminal residue (Thr198), and this appeared to stabilize the protein, thus increasing its expression and causing cell cycle arrest as well as appearing to promote autophagy. Phosphorylation of Thr198 increased in response to AICAR treatment or glucose starvation of cells. Although some evidence was presented that Thr198 is directly phosphorylated by AMPK, the site is not a perfect fit to the AMPK recognition motif (for example, being the C-terminal residue, there is no hydrophobic residue at P+4), and mutation of Thr198 only had a modest effect on phosphorylation by AMPK in cell-free assays [156]. Further studies are therefore required to confirm that this is a direct effect of AMPK.

Although AMPK activation by both AICAR [14] and low glucose [60] requires the presence of LKB1, AMPK could still cause G1 arrest in three different LKB1-deficient tumour cell lines if it was activated by the addition of a Ca^2+^ ionophore to activate the alternative upstream kinase, CaMKK2. This effect was abolished either by expression of a dominant negative AMPK-α2 mutant or by a double knockout of AMPK-α1 and -α2 [157]. Thus, AMPK can cause G1 arrest even in the absence of its tumour suppressor upstream kinase, LKB1. Interestingly, treatment of cells with the Ca^2+^ ionophore A23187 caused G1 arrest without affecting the expression of CDKN1A or CDKN1B, despite the fact that the overall expression of both was reduced by expression of the dominant negative mutant or the double knockout [157]. Thus, in this case changes in CDKN1A or CDKN1B expression cannot be the sole explanation for cell cycle arrest.

## 5. AMPK and cancer—evidence from mouse models

We will now discuss the evidence that, depending upon the context, AMPK can act either as a tumour suppressor or as a tumour promoter in mouse models.

### 5.1. AMPK—a tumour suppressor?

With the discovery that AMPK activation requires the tumour suppressor LKB1, the realization that AMPK inhibits cell growth and proliferation, and the epidemiological evidence that the AMPK activator, metformin, provides protection against cancer, it seemed increasingly likely that AMPK would also be a tumour suppressor. One caveat was that, soon after the discovery that LKB1 acted upstream of AMPK, LKB1 was found to be required for the phosphorylation and activation of at least 12 other kinases closely related to AMPK (now referred to as the *AMPK-related kinase* or *ARK* family), which share very similar sequences within their activation loops [158,159]. Although none of the ARKs (unlike AMPK) are known to inhibit cell growth and proliferation, knockdown of LKB1 using RNAi was reported to enhance expression of SNAIL, a protein that promotes the epithelial-to-mesenchymal transition, and hence metastasis of tumour cells, by reducing the phosphorylation of DIXDC1 by two of the ARKs, MARK1 and MARK4 [160]. It therefore remains possible that at least some of the tumour suppressor effects of LKB1 might be mediated by one or more of the ARKs, rather than AMPK.

Confirmation of a tumour suppressor role for AMPK *in vivo* required the study of tumorigenesis in AMPK knockout mice. However, there are two isoforms of the catalytic subunit (α1 and α2) and a global double knockout is embryonic lethal [161], thus necessitating the use of tissue-specific double knockouts. While this approach is now possible, it is also very time-consuming. However, a shortcut arose with the realization that cells of the haematopoietic lineage, including lymphocytes, exclusively express AMPK-α1 [162]. Thus, to study the role of AMPK in lymphomas and/or leukaemias, it was only necessary to knock out a single gene, i.e. the *Prkaa1* gene encoding AMPK-α1.

The first study to suggest that AMPK was a tumour suppressor used a Eµ-Myc model [163], in which B-cell lymphoma is induced by transgenic over-expression of the *Myc* oncogene from a B-cell-specific promoter. Consistent with the idea that AMPK-α1 is a tumour suppressor, loss of both alleles of *Prkaa1* in this model markedly accelerated development of B-cell lymphomas, whereas loss of a single allele had an intermediate effect. Eµ-Myc lymphoma cells and other tumour cells expressing shRNAs targeted at AMPK-α1 were also studied *in vitro*. In general, the AMPK knockdown cells exhibited mTORC1 hyper-activation and increased glucose uptake and lactate production compared with controls, and this appeared to be due to increased expression of *hypoxia-inducible transcription factor-1α* (*HIF*-*1α*) [163]. The 5′-UTR of mRNA encoding HIF-1α contains 5′-TOP sequences [164] and their translation is thus enhanced by mTORC1 activation ([165]; see §4.2). Thus, loss of AMPK in the tumour progenitor cells enhances glucose uptake and glycolysis even under normoxic conditions. This is an example of the well-known ‘Warburg effect’, in which tumour cells display high levels of glucose consumption in order to generate precursors for biosynthesis derived from the pentose phosphate pathway and glycolysis. For example, ribose-5-phosphate for nucleotide biosynthesis and NADPH for lipid synthesis are generated via the pentose phosphate pathway, while serine (required for one-carbon metabolism used in purine nucleotide biosynthesis) is generated by a pathway that branches off from the glycolytic intermediate 3-phosphoglycerate [126].

A drawback with this B-cell lymphoma model was that *Prkaa1* was knocked out globally [163], so it was not possible to conclude that the effect was due to a cell-autonomous loss of AMPK-α1 in the B-cell progenitors themselves, rather than an indirect effect of loss of AMPK-α1 in some other cell type. In an attempt to address this, wild-type mice were irradiated to inactivate their endogenous immune system, and were then reconstituted with haematopoietic stem cells from either Eµ-Myc/*Prkaa1^−/−^* or Eµ-Myc/*Prkaa1^+/+^* mice. Interestingly, all of the mice receiving AMPK knockout cells developed lymphomas, but only 20% of those receiving the AMPK wild-type cells [163], thus supporting the idea that the effect of AMPK loss was at least partly cell autonomous.

Another study involved crossing mice with global knockouts of the genes encoding p53 (*Trp53*) and AMPK-β1 (*Prkab1*), the latter being the principal β subunit isoform expressed in T-cell precursors in the thymus [166]. Knockout of *Prkab1* caused earlier onset of T-cell lymphomas in both homozygous and heterozygous p53 knockouts, suggesting that β1 had a tumour suppressor role in T-cell lymphoma. However, once again the knockout of *Prkab1* in this model was global rather than T-cell specific, so it was not possible to conclude whether this was a cell-intrinsic effect on AMPK in the tumour progenitor cells themselves.

**Figure 5.**
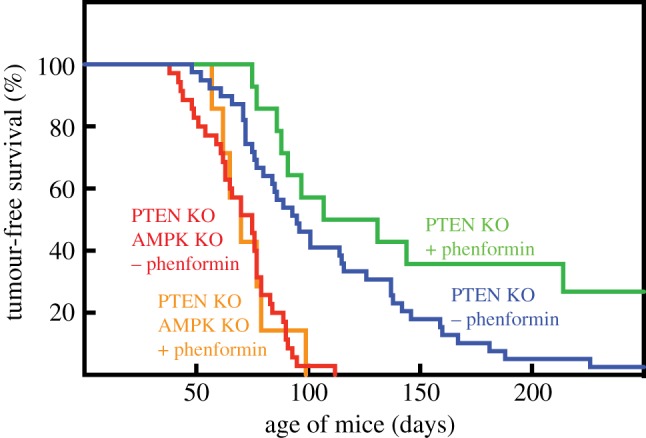
Effect of T-cell knockout of AMPK (AMPK KO) and oral phenformin on tumour-free survival in mice bearing T-cell knockout of PTEN (PTEN KO). Where indicated, phenformin was administered in drinking water starting from 30 days of age. Original data from [167].

A specific loss of AMPK in the tumour progenitor cells has recently been achieved using a model of T-cell acute lymphoblastic leukaemia/lymphoma (T-ALL) [167]. As reported previously [168], mice with a T-cell-specific knockout of PTEN (using Cre recombinase expressed from the *Lck* promoter) started to develop lymphomas at about 50 days of age, and essentially all of the mice had developed T-ALL by 150 days. While knocking out the *Prkaa1* gene using the same *Lck*-Cre system had no effect on its own, when combined with PTEN knockout the lymphomas arose earlier and overall tumour-free survival was greatly reduced (figure 5) [167]. These results suggested that basal AMPK activity in developing T cells is sufficient to provide protection against T-ALL. However, this model also provided an excellent opportunity to test whether treatment with biguanides would protect against this type of cancer (see §3.3). Since the expression of AMPK-α1 would be absent in lymphoma cells and their progenitors but normal everywhere else, it would also be possible to determine whether any effect of biguanides was a cell-intrinsic effect to activate AMPK in the tumour progenitor cells themselves. Rather disappointingly, metformin had no effect, which correlated with a lack of AMPK activation and a failure to detect metformin by liquid chromatography–mass spectrometry (LC:MS) in the thymus of mice with lymphomas. By contrast, phenformin significantly enhanced tumour-free survival, and this correlated with AMPK activation, and detection of phenformin by LC:MS, in the thymus of mice with lymphoma. Intriguingly, protection against T-ALL by phenformin was only observed when the tumours expressed AMPK, with no effect in the AMPK knockouts (figure 5). Thus, protection against T-ALL by phenformin was dependent upon the expression of AMPK in the tumour progenitor cells, and was cell autonomous, while the failure of metformin to provide protection was due to lack of uptake of the drug by thymocytes. Phenformin has also been shown recently to slow growth of murine breast cancer cells *in vivo* in a mouse allograft model, although the role of AMPK was not examined [169].

Another mouse model suggesting a tumour suppressor role for AMPK used prostate epithelial-specific knockouts of the *Pten* and *Prkab1* genes [170]. Although the knockout of *Prkab1* as well as *Pten* did not affect prostate size, it did result in a higher proliferative index and pathological grade. A drawback with this model was that the prostate gland also expresses AMPK-β2, which might have partially compensated for lack of β1 and might be why the effects on tumorigenesis were relatively modest.

Other evidence supporting the idea that AMPK is a tumour suppressor comes from studies of ubiquitin ligases involved in cellular degradation of AMPK subunits. MAGE-A3/-A6 are closely related members of the *melanoma antigen* family of proteins, which are normally only expressed in testis but become re-expressed in many tumours, hence their designation as *tumour antigens* [171]. MAGE-A3/-A6 bind to the ubiquitin E3 ligase TRIM28, and a screen revealed AMPK-α1 to be a target for polyubiquitylation by this complex, with consequent proteasomal degradation. Consistent with this, knockdown of MAGE-A3/A6 or TRIM28 in tumour cells increased the expression of AMPK-α1 and triggered the expected changes in metabolism and signalling, including inhibition of mTORC1. Finally, various human tumour cells that express MAGE-A3/-A6 have reduced levels of AMPK-α1 protein [171].

Another cancer-associated ubiquitin ligase, UBE2O, targets degradation of α2, the other catalytic subunit isoform of AMPK [172]. Knockout of *Ube2o* attenuated tumour development in mouse models of both breast and prostate cancer, supporting the idea that the protein has tumour-promoting functions. A search identified AMPK-α2 as an UBE2O-interacting protein that is targeted for polyubiquitylation and proteasomal degradation, and the levels of α2 but not α1 were upregulated in tissues from *Ube2o^−/−^* mice. A human colon carcinoma cell line also grew less rapidly in mouse xenografts when UBE2O was knocked down using shRNA, and this was reversed by concurrent knockdown of AMPK-α2 but not -α1. The *UBE2O* gene is located in humans at 17q25, a region amplified in up to 20% of breast, bladder, liver and lung carcinomas. Using immunohistochemistry of human breast tumours, there was a negative correlation between expression of UBE2O and AMPK-α2, but a positive correlation between UBE2O expression and S6 phosphorylation, a marker for the mTORC1 pathway [172].

### 5.2. AMPK—a tumour promoter?

Despite the evidence discussed in the previous section that AMPK-α1 and -β1 are tumour suppressors that protect against the development of B- and T-cell lymphomas as well as prostate cancer, other studies suggest that AMPK may protect the tumour cells (rather than the patient), and thus *promote* tumour formation, at least when disease is already established. Rathmell's group used a different model of T-ALL in which oncogenic NOTCH1 was expressed *in vitro* in murine haematopoietic stem cells that carried a floxed AMPK-α1 gene and Cre recombinase driven by a tamoxifen-inducible promoter. These were multiplied in irradiated mice, and then injected into irradiated secondary recipient mice. After a period of 10 days to allow disease to become established, the mice were then treated with tamoxifen to acutely delete AMPK-α1 in the T-ALL cells. In this model, knocking out AMPK-α1 reduced the recovery of T-ALL cells in spleen, lymph nodes and bone marrow, and enhanced survival of the mice [173]. Thus, once T-ALL tumour cells have developed the presence of AMPK-α1 appears to *enhance* T-ALL cell viability and reduce mouse survival. While AMPK therefore acts as a tumour suppressor during the development of T-ALL [167], once the tumours have occurred it appears to paradoxically switch to being a tumour promoter instead.

Another study using a mouse model of acute myeloid leukaemia (AML) also concluded that AMPK acted as a tumour promoter [174]. Here, mice carrying floxed alleles of *Prkaa1* and *Prkaa2,* as well as Cre recombinase expressed from the *Mx1* promoter, were injected with poly(I:C) to delete AMPK-α1 and -α2 from haematopoietic cells, with mice lacking *Mx1-Cre* as controls. Haematopoietic progenitor cells from these mice were then transduced with retroviruses expressing three different cancer-promoting gene fusions (MLL-AFP, MOZ-TIF2 or BCR-ABL) and were then transplanted into irradiated recipient mice. The absence of AMPK from the leukaemia-initiating cells either delayed the onset of disease (BCR-ABL) or enhanced mouse survival (MLL-AFP or MOZ-TIF1). Thus, the presence of AMPK was required to maintain full leukaemogenic potential of the cells in these models. Evidence was provided that this was because the lack of AMPK increased the recovery of reactive oxygen species (ROS) in leukaemia-initiating cells from bone marrow, correlating with decreased ratios of reduced : oxidized NADP and glutathione, and increased DNA damage. This was ascribed to a reduced glucose uptake via GLUT1, which is regulated by AMPK via phosphorylation of TXNIP (see §4.1). The authors also proposed that the leukaemia-initiating cells lacking AMPK were particularly vulnerable to stress in the bone marrow, because the glucose concentrations were lower than in peripheral blood, especially under conditions of dietary restriction of the mice [174].

Consistent with these findings, reduced survival of AMPK-deficient human tumour cells undergoing stress has been observed in several *in vitro* studies. For example, LKB1-null tumour cells, or LKB1-expressing tumour cells with AMPK-α1 knocked down using shRNA, were more susceptible to cell death induced by glucose starvation or extracellular matrix detachment, suggesting that AMPK activation protected against these insults [175]. In another example, a synthetic lethal siRNA screen was carried out to detect protein kinases required for survival of U2OS cells that over-expressed the Myc oncogene from a tamoxifen-inducible promoter. One of the top hits was AMPK-α1, which was also shown to be activated during Myc over-expression [176].

Evidence that AMPK can promote tumours was also obtained recently using a mouse model of lung cancer in which the tumours develop *in situ* at their site of origin, and in which the authors had ‘bitten the bullet’ by knocking out both AMPK-α1 and -α2. Here, mice expressing Lox-STOP-Lox alleles of the KRAS^G12D^ oncogene and firefly luciferase were crossed with mice expressing floxed alleles of *Tp53* (encoding p53) and/or *Stk11* and/or *Prkaa1* plus *Prkaa2*. To model non-small cell lung carcinoma, Cre-recombinase was delivered to the lungs by nasal inhalation of lentiviral vectors. This procedure triggers recombination at twin loxP sites in a small subset of lung epithelial cells, in which expression of KRAS^G12D^ and luciferase would be switched on, and p53 and/or LKB1 and/or AMPK-α1/-α2 would be knocked out; expression of luciferase also allowed tumours to be imaged by bioluminescence, and thus their growth to be monitored *in vivo*. Knockout of LKB1 enhanced growth in tumours expressing mutant K-Ras as reported previously [177] but, by contrast, knockout of both AMPK-α1 and -α2 was found to cause *reductions* in the size and number of lung tumours, especially in tumours expressing mutant K-Ras and lacking p53. Overall, these results confirmed that LKB1 is a tumour suppressor in non-small cell lung cancer as expected, while the presence of either AMPK-α1 or -α2 *promoted* tumour growth [178].

## 6. Evidence from analysis of human cancer genomes

**Figure 6.**
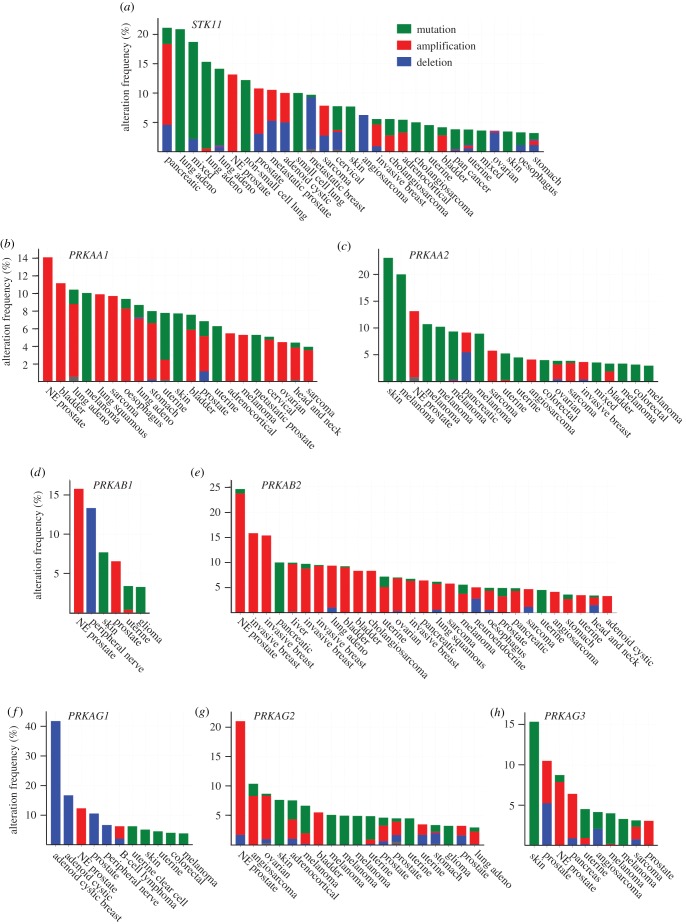
Summary of genetic alterations in human cancer in genes encoding (*a*) LKB1 (*STK11*), and (*b–h*) the seven genes encoding AMPK subunit isoforms. Based on analysis of the ‘curated set of non-redundant studies’ in the cBioPortal database in early April 2019, using the gene names shown.

**Figure 7.**
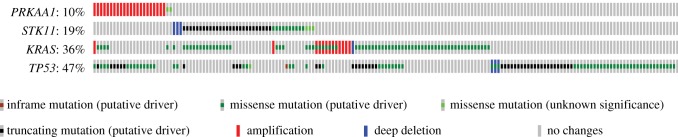
Co-occurrence or mutual exclusion of genetic alterations in the *PRKAA1* (AMPK-α1), *STK11* (LKB1), *KRAS* (K-Ras) and *TP53* (p53) genes in human lung adenocarcinoma. Results were generated using cBioPortal from the results of a single study [181]. Each column of vertically aligned bars represents a single case; cases with no alterations in any of the genes are not shown.

Although most of the evidence discussed in §5 was obtained in mouse models of cancer, comparison of genetic alterations in genes encoding the LKB1-AMPK pathway in biopsies of human cancers, compared with normal tissue, can also provide useful clues about roles of the pathway in human cancer. The cBioPortal database (http://www.cbioportal.org/ [179,180]) provides a particularly user-friendly way to analyse the many studies of human cancer of this type that have been performed to date. Figure 6 summarizes genetic changes in the *STK11* gene encoding LKB1, and all seven genes encoding subunit isoforms of AMPK, extracted from cBioPortal in April 2019. Each vertical bar represents an individual cancer genome project, with the height of the bar representing the percentage of cases where genetic alterations were seen (only studies with changes in greater than or equal to 3% of cases are shown). Since LKB1 is a known tumour suppressor, one would expect to observe mainly mutations (green bars) or deletions (blue bars) when analysing *STK11*. This is indeed generally the case (figure 6*a*), although there are some anomalous cancer studies where gene amplification was observed instead (red bars), particularly in pancreatic and prostate cancers. By contrast, changes in the *PRKAA1* gene, encoding AMPK-α1, were mostly amplifications (note preponderance of red bars in figure 6*b*), which is more consistent with the idea that AMPK-α1 can act as a tumour promoter. An important caveat is that gene amplifications in cancer usually involve whole segments of chromosomes rather than individual genes. It was therefore possible that the *PRKAA1* gene is located close to an oncogene for which amplification was being selected, with *PRKAA1* simply accompanying it as an innocent bystander. However, an argument against that possibility comes from analysis of concurrent genetic changes in *STK11* and *PRKAA1* in single cancer studies, such as the 230 cases of lung adenocarcinoma in The Cancer Genome Atlas (figure 7) [181]. In that study, the *STK11* gene was either deleted or mutated (mostly truncations or missense mutations predicted to cause loss of function) in 43 cases (19%) and *PRKAA1* was amplified in 22 (10%). However, these changes never coincided (*p* = 0.005), which would be expected to occur by random chance if they were occurring independently. The most frequent mutations in this study of lung adenocarcinoma were in the *KRAS* (36%) and *TP53* genes (47%), encoding K-Ras and p53. Interestingly, amplification of *PRKAA1* was almost mutually exclusive with mutations in *KRAS* (*p* = 0.005), but co-occurred with mutations in *TP53* (*p* < 0.001).

Why should amplification of the AMPK-α1 gene be mutually exclusive with mutations in the LKB1 gene? The answer to this seems obvious, because there would be little point in over-expressing AMPK-α1 if LKB1 was not present to phosphorylate and activate it. These considerations suggest that *PRKAA1* amplification is being selected for, rather than just being an innocent bystander. However, why amplification of the AMPK-α1 gene should co-occur with mutations in p53 is less obvious. The classical role of p53 [182] is to become stabilized or activated in response to DNA damage, and to cause a G1 cell cycle arrest in order to allow time for the damage to be repaired, which it achieves by inducing transcription of genes such as the G1 cyclin-dependent kinase inhibitor p21^CIP1^ (CDKN1A). Intriguingly, as already discussed in §2.5, AMPK complexes containing α1 are also activated by genotoxic agents such as etoposide, and can trigger a similar G1 cell cycle arrest [11]. It therefore seems possible that *PRKAA1* amplification may be selected for in p53-null tumours because over-expression of AMPK-α1 can compensate to some extent for p53 loss, and could thus enhance survival of p53-null tumour cells undergoing genotoxic stress.

In marked contrast to the frequent amplification of the *PRKAA1* gene in cancer, the *PRKAA2* gene encoding AMPK-α2 is much more often mutated (note preponderance of green bars in figure 6*c*). Interestingly, all six of the cancer studies where the gene was most frequently mutated (10–23% of cases) were of skin cancer or melanoma. The reasons for this are not clear, but separate analysis showed that in all of the skin cancer/melanoma studies listed in cBioPortal there were 80 mutations affecting AMPK-α2 and just 10 affecting α1. Although it is not yet clear how many of the former cause loss of function in α2 complexes, these results suggest that AMPK-α2 may play a tumour suppressor role in skin cancer and melanoma.

When it comes to the AMPK-β subunits, there was a striking difference between the behaviour in human cancers of the *PRKAB1* and *PRKAB2* genes, encoding β1 and β2 (figure 6*d*,*e*). While genetic changes in *PRKAB1* were detected in just a very small number of cancer studies and were quite variable in genetic type, the *PRKAB2* gene was frequently amplified in numerous different cancers (note preponderance of red bars in figure 6*e*), suggesting, if anything, a tumour promoter role. Since the C-terminal domain of the β subunit (β-CTD) forms the ‘core’ of the heterotrimeric AMPK complex (see §2.2), over-expression of β2 may perhaps help to stabilize and increase expression of the α and γ subunits, even when the genes encoding those subunits lack genetic alterations. However, why it should only be the gene encoding β2, and not β1, that is amplified remains unclear.

Alterations in the genes encoding the three γ subunits tend to occur at a lower frequency than those encoding the α and β subunits, and are more mixed in genetic type (figure 6*f–h*). However, there were some interesting findings, such as the 41% of cases (albeit only five out of 12) in which the *PRKAG1* gene was deleted in adenoid cystic breast cancer [183].

Looking at the genetic alterations occurring in the genes encoding LKB1 and AMPK subunits in human cancer, one striking observation is that all eight genes are frequently amplified in neuroendocrine prostate cancer (labelled NE prostate in figure 6). This is a subset of prostate cancer that has become resistant to anti-androgen treatment [184]. The significance of this intriguing observation remains unclear at present.

## 7. Conclusion—is AMPK a tumour suppressor or a tumour promoter, or both?

In this final section we will attempt to reconcile the apparently conflicting reports that AMPK can variously act to promote or suppress tumorigenesis. Our view is that AMPK can act either as a tumour suppressor or a tumour promoter, depending on the context. It can be argued that in all of the mouse studies where a tumour suppressor role was supported (e.g. in the Eµ-Myc model of B-cell lymphoma [163], the p53-null [166] and PTEN-null [167] models of T-cell lymphoma and the PTEN-null model of prostate cancer [170]), AMPK function had been knocked out *prior to tumorigenesis*. For example, in the Eµ-Myc model [185], loss of AMPK-α1 would have occurred during embryogenesis whereas, although over-expression of Myc in pre-B cells has certainly occurred by 35–50 days of age [186], lymphomas do not start to arise until 50 days and their median onset is ≈80 days [187]. Thus, events additional to Myc over-expression must occur before lymphomas are generated. The same applies to the PTEN knockout model of T-ALL, where the *Lck* promoter-driven knockout of PTEN and/or AMPK-α1 would have occurred by 30 days of age but lymphomas did not start to arise until later (figure 5).

On the other hand, in those mouse models of cancer where AMPK appeared to be acting as a tumour promoter, it can be argued that the knockout of AMPK usually occurred either simultaneous with, or even *after*, tumorigenesis had been initiated. For example, in the study of T-ALL by Kishton *et al.* [173] (§5.2), transformation was generated *in vitro* by forced expression of an oncogenic mutant of Notch1, and the T-ALL cells were then transferred to irradiated recipient mice and disease allowed to become established prior to AMPK being knocked out by treatment with tamoxifen. It is particularly instructive to compare this model with our own more recently published model of T-ALL [167], where AMPK-α1 had been specifically knocked out in T-cell progenitors prior to lymphomas starting to occur, in which basal AMPK was clearly protecting against development of lymphomas, and in which activation of AMPK using phenformin provided further protection.

Coming to other mouse studies that support a tumour promoter role for AMPK, in the autochthonous model of non-small cell lung cancer [178], knockout of AMPK would have occurred simultaneously with expression of mutant K-Ras and loss of p53, which may have been sufficient to trigger tumorigenesis on their own. The only study that supported a tumour promoter role but where AMPK had been knocked out *prior* to disease onset was the model of AML by Saito *et al.* [174]. However in that case (as in the study of T-ALL by Kishton *et al.* [173]) transformation had been achieved by enforced expression of oncogenes *in vitro* in haematopoietic progenitor cells, and the real test of the role of AMPK was in the survival and/or proliferation of the leukaemia cells *in vivo* in irradiated recipient mice. It can be argued that these two studies, by carrying out transformation *in vitro*, may have been less likely to detect a tumour suppressor role of AMPK.

Overall we propose that, when loss of AMPK occurs prior to initiation of tumorigenesis *in vivo,* this would remove the restraints on the mTORC1 pathway and unleash other biosynthesis processes and the cell cycle, thus transforming the cells into a metabolic and proliferative state that is primed for tumour formation. Under these circumstances, AMPK acts as a tumour suppressor, and AMPK activators may provide additional protection against tumorigenesis, such as the effect of phenformin in T-ALL [167]. These results suggest that AMPK activators might one day find a place in providing protection against cancer, perhaps in individuals who are at high risk of developing the disease. If biguanides are used, it might also make sense to use phenformin which, being more membrane permeable than metformin even in the absence of a transporter, is much more likely to activate AMPK in the tumour progenitor cells. Although phenformin was withdrawn for treatment of type 2 diabetes because of the risk of life-threatening lactic acidosis, the risk of this complication was actually quite low (≈64 cases per 100 000 patient-years [188]), and might be more acceptable in the context of cancer rather than diabetes. Alternatively, some of the other AMPK activators discussed in §3 might perhaps be developed for this purpose.

We also propose that, once the cancer cells have started to grow *in vivo*, AMPK switches from being a tumour suppressor to a tumour promoter (like the transformation of the benevolent Dr Jekyll into the malevolent Dr Hyde in Stevenson's novel!). Under these circumstances, the role of AMPK is to protect the cell in which it is expressed, irrespective of whether that cell is a cancer cell or a normal cell. By protecting cancer cells against stresses such as shortage of oxygen or nutrients, or oxidative or genotoxic stress, AMPK would enhance their survival and thus, in the long term, promote growth of tumours. Under these circumstances, AMPK is acting as a tumour promoter, which suggests that AMPK inhibitors might be efficacious in treatment of cancer. They may be particularly effective: (i) in cases where the *PRKAA1* or *PRKAB2* genes are amplified, causing AMPK over-expression; (ii) when given in combination with genotoxic treatments such as etoposide or radiotherapy, thus reducing the viability of tumour cells during such therapies. At present we do not have any well-characterized and specific inhibitors of AMPK (see §3.4), but future work can be directed at correcting that deficiency.

## Supplementary Material

Reviewer comments

## Data accessibility

This article has no additional data.
