# Supplementary material for: The strange case of AMPK and cancer: Dr Jekyll or Mr Hyde?
Source: Open Biol. 2019 Jul 10;9(7):190099. doi: 10.1098/rsob.190099 (PMC6685927; doi:10.1098/rsob.190099)
Supplement: Reviewer comments [file rsob190099_review_history.pdf]

THE ROYAL SOCIETY
PUBLISHING

OPEN
BIOLOGY

The strange case of AMPK and cancer: Dr Jekyll or Mr Hyde?

Diana Vara-Ciruelos, Fiona M. Russell and D. Grahame Hardie

Article citation details
Open Biol. **9**: 190099.
http://dx.doi.org/10.1098/rsob.190099

Review timeline
Original submission: 3 May 2019
Revised submission: 3 June 2019
Final acceptance: 11 June 2019

Note: Reports are unedited and appear as submitted by the referee. The review history appears in chronological order.

Review History

RSOB-19-0099.R0 (Original submission)

Review form: Reviewer 1

Recommendation
Accept with minor revision (please list in comments)

Are each of the following suitable for general readers?

a) **Title**
Yes

b) **Summary**
Yes

c) **Introduction**
Yes

Is the length of the paper justified?
Yes

Reports © 2019 The Reviewers; Decision Letters © 2019 The Reviewers and Editors; Responses © 2019 The Reviewers, Editors and Authors. Published by the Royal Society under the terms of the Creative Commons Attribution License http://creativecommons.org/licenses/by/4.0/, which permits unrestricted use, provided the original author and source are credited

Should the paper be seen by a specialist statistical reviewer?
No

Is it clear how to make all supporting data available?
Not Applicable

Is the supplementary material necessary; and if so is it adequate and clear?
No

Do you have any ethical concerns with this paper?
No

Comments to the Author
A well written review of the field by a group that contributed much to current knowledge.

One can always discuss decisions regarding scope in this kind of review, but since the authors decided to include discussion of biguanides as indirect activators of AMPK in the context of neoplasia, two recent papers would add to this aspect of the discussion: Lord et al Cell Metab. 2018 Nov 6;28(5):679-688 recently reported a pharmacodynamic study of metformin effects on breast cancer in patients, revealing effect sizes smaller than those seen in preclinical models (emphasizing relevance of pharmacokinetics) and Hulea et al Cell Metab. 2018 Dec 4;28(6):817-832 show how metabolic plasticity of neoplastic cells can influence response to biguanides.

I have no major criticisms.

Decision letter (RSOB-19-0099.R0)

02-Jun-2019

Dear Dr Hardie

We are pleased to inform you that your manuscript RSOB-19-0099 entitled "The strange case of AMPK and cancer: Dr Jekyll or Mr Hyde?" has been accepted by the Editor for publication in Open Biology. The reviewer has recommended publication, but also suggest some minor revisions to your manuscript. Therefore, we invite you to respond to the reviewer's comments and revise your manuscript.

Please submit the revised version of your manuscript within 7 days. If you do not think you will be able to meet this date please let us know immediately and we can extend this deadline for you.

To revise your manuscript, log into https://mc.manuscriptcentral.com/rsob and enter your Author Centre, where you will find your manuscript title listed under "Manuscripts with Decisions." Under "Actions," click on "Create a Revision." Your manuscript number has been appended to denote a revision.

You will be unable to make your revisions on the originally submitted version of the manuscript. Instead, please revise your manuscript and upload a new version through your Author Centre.

When submitting your revised manuscript, you will be able to respond to the comments made by the referee(s) and upload a file "Response to Referees" in "Section 6 - File Upload". You can use

this to document any changes you make to the original manuscript. In order to expedite the processing of the revised manuscript, please be as specific as possible in your response to the referee(s).
Please see our detailed instructions for revision requirements https://royalsociety.org/journals/authors/author-guidelines/.

Before uploading your revised files please make sure that you have:

1) A text file of the manuscript (doc, txt, rtf or tex), including the references, tables (including captions) and figure captions. Please remove any tracked changes from the text before submission. PDF files are not an accepted format for the "Main Document".

2) A separate electronic file of each figure (tiff, EPS or print-quality PDF preferred). The format should be produced directly from original creation package, or original software format. Please note that PowerPoint files are not accepted.

3) Electronic supplementary material: this should be contained in a separate file from the main text and meet our ESM criteria (see https://royalsociety.org/journals/authors/author-guidelines/). All supplementary materials accompanying an accepted article will be treated as in their final form. They will be published alongside the paper on the journal website and posted on the online figshare repository. Files on figshare will be made available approximately one week before the accompanying article so that the supplementary material can be attributed a unique DOI.

Online supplementary material will also carry the title and description provided during submission, so please ensure these are accurate and informative. Note that the Royal Society will not edit or typeset supplementary material and it will be hosted as provided. Please ensure that the supplementary material includes the paper details (authors, title, journal name, article DOI). Your article DOI will be 10.1098/rsob.2016[last 4 digits of e.g. 10.1098/rsob.20160049].

4) A media summary: a short non-technical summary (up to 100 words) of the key findings/importance of your manuscript. Please try to write in simple English, avoid jargon, explain the importance of the topic, outline the main implications and describe why this topic is newsworthy.

Images
We require suitable relevant images to appear alongside published articles. Do you have an image we could use? Images should have a resolution of at least 300 dpi, if possible.

Data-Sharing
It is a condition of publication that data supporting your paper are made available. Data should be made available either in the electronic supplementary material or through an appropriate repository. Details of how to access data should be included in your paper. Please see http://royalsocietypublishing.org/site/authors/policy.xhtml#question6 for more details.

Data accessibility section
To ensure archived data are available to readers, authors should include a 'data accessibility' section immediately after the acknowledgements section. This should list the database and accession number for all data from the article that has been made publicly available, for instance:
• DNA sequences: Genbank accessions F234391-F234402
• Phylogenetic data: TreeBASE accession number S9123
• Final DNA sequence assembly uploaded as online supplemental material
• Climate data and MaxEnt input files: Dryad doi:10.5521/dryad.12311

Once again, thank you for submitting your manuscript to Open Biology, we look forward to receiving your revision. If you have any questions at all, please do not hesitate to get in touch.

Sincerely,
The Open Biology Team
mailto:openbiology@royalsociety.org

Reviewer's Comments to Author:
Referee:
Comments to the Author(s)
A well written review of the field by a group that contributed much to current knowledge.

One can always discuss decisions regarding scope in this kind of review, but since the authors decided to include discussion of biguanides as indirect activators of AMPK in the context of neoplasia, two recent papers would add to this aspect of the discussion: Lord et al Cell Metab. 2018 Nov 6;28(5):679-688 recently reported a pharmacodynamic study of metformin effects on breast cancer in patients, revealing effect sizes smaller than those seen in preclinical models (emphasizing relevance of pharmacokinetics) and Hulea et al Cell Metab. 2018 Dec 4;28(6):817-832 show how metabolic plasticity of neoplastic cells can influence response to biguanides.

I have no major criticisms.

Author's Response to Decision Letter for (RSOB-19-0099.R0)

See Appendix A.

Decision letter (RSOB-19-0099.R1)

11-Jun-2019

Dear Dr Hardie

We are pleased to inform you that your manuscript entitled "The strange case of AMPK and cancer: Dr Jekyll or Mr Hyde?" has been accepted by the Editor for publication in Open Biology.

You can expect to receive a proof of your article from our Production office in due course, please check your spam filter if you do not receive it within the next 10 working days. Please let us know if you are likely to be away from e-mail contact during this time.

Thank you for your fine contribution. On behalf of the Editors of Open Biology, we look forward to your continued contributions to the journal.

Sincerely,

The Open Biology Team
mailto: openbiology@royalsociety.org

Appendix A

We are please that the reviewer says that this is “a well written review of the field by a group that contributed much to current knowledge” and “I have no major criticisms”. In response to their suggestion to cite two recent papers on the role of biguanides in cancer treatment, we have now cited Lord et al (2018), along with three other papers, on lines 384-386, and Hulea et al (2018) on lines 690-692.
